# Extranodal natural killer/T-cell lymphoma nasal type with extensive ocular tissue involvement: a case report

**DOI:** 10.1186/s13000-021-01167-3

**Published:** 2021-11-11

**Authors:** Ruoan Han, Yang Jiang, Ailing Bian, Zhilan Meng, Hao Chen, Junjie Ye

**Affiliations:** 1 Department of Ophthalmology, Peking Union Medical College Hospital, Key Laboratory of Ocular Fundus Diseases, Chinese Academy of Medical Sciences, Beijing, 100730 China; 2 Department of Pathology, Peking Union Medical College Hospital, Chinese Academy of Medical Sciences, Beijing, 100730 China; 3 Department of Pathology, The Second Affiliated Hospital of Guangxi Medical University, Guangxi, 530005 China

**Keywords:** Extranodal natural killer/T-cell lymphoma, Ocular tissue involvement, Ciliary body, Case report

## Abstract

**Background:**

To report a rare case of extranodal natural killer/T-cell lymphoma (ENKTL), nasal type related to extensive ocular tissue, including conjunctiva, ciliary body, vitreous and retina.

**Case presentation:**

A 52-year-old woman who had been treated by radiotherapy for ENKTL, nasal type in the right nasal cavity presented with a dramatic deterioration of vision in right eye. Physical and accessory examination showed extensive ocular tissue related, including conjunctiva, ciliary body, vitreous and retina. Vitreous specimen and conjunctiva biopsy revealed the presence of ENKTL, nasal type in the right eye. She was treated with systemic and ophthalmic chemotherapy, her ocular symptoms significantly improved, and systemic condition remained stable 7 months after the diagnosis.

**Conclusions:**

Extranodal natural killer/T-cell lymphoma, nasal type is an aggressive disease and may relate extensive ocular tissue and course dramatic vision deterioration. It is important to observe ocular related and begin aggressive combined therapy as early as possible after diagnosis.

## Background

Extranodal natural killer/T-cell lymphoma (ENKTL), nasal type is a form of lymphoma with an immature natural killer (NK)-cell phenotype. Most of the cases involve the nasopharyngeal region, while other extranasal regions can also be involved. ENKTL involving ocular or ocular adnexa tissues are rare. Here, we report a case of nasal ENKTL related to ocular tissue, including conjunctiva, ciliary body, vitreous and retina.

## Case presentation

**Fig. 1:**
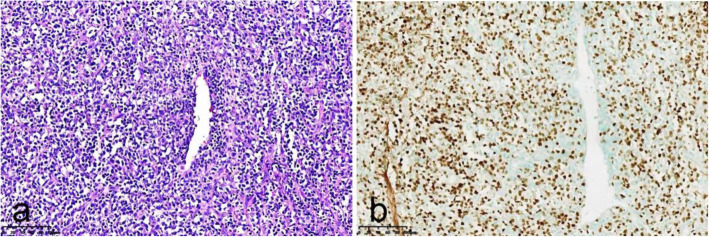
Photomicrographs of nasal biopsy. a: Histologic photomicrograph of nasal biopsy (hematoxylin-erosin; × 20) showing fragmented tumor tissue. b: Immunohistochemical photomicrograph of nasal biopsy (EBER; × 20)

**Fig. 2:**
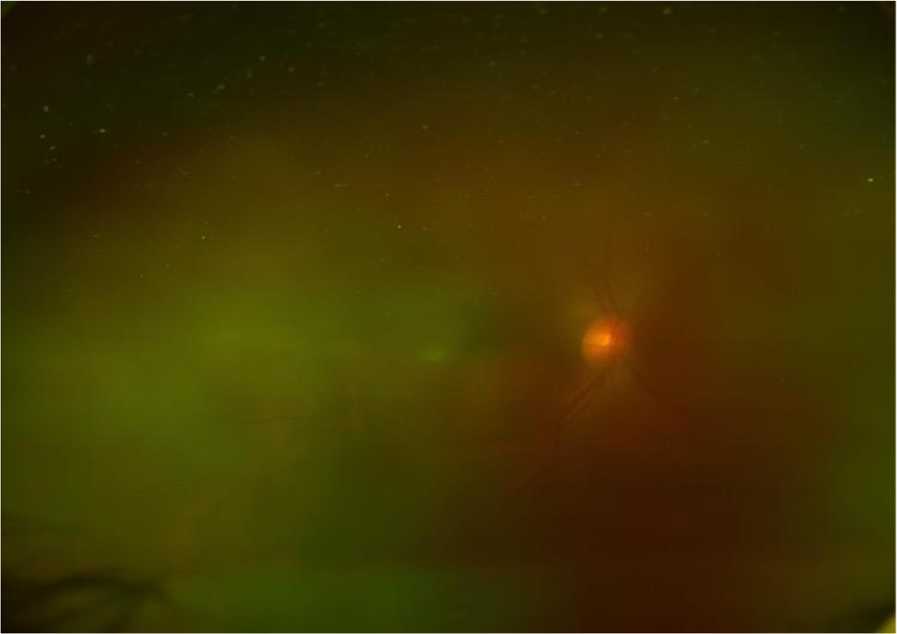
Ultra-wide-field fundus image of right eye showed almost normal. BCVA was 20/50 of right eye at that time

A 52-year-old woman presented in June 2020 with a dramatic deterioration of vision in her right eye. She presented with a right nasal cavity mass in April 2020 and was diagnosed as NK/T-cell lymphoma nasal type. The nasal biopsy showed fragmented tumor tissue, most of the surface without epithelial coverage, moderately large dysmorphic lymphocytes in the stroma diffusely and consistently proliferated and infiltrated, with irregular nuclei, hollow cytoplasm, visible vascular wall invasion, with necrotic exudation. Immunohistology of nasal biopsy was positive for CD3, CD56, Ki67 80%, TIA1, granzyme B and Epstein-Barr virus (EBV), but negative for CD20, PAX5, CD30, T-bet, CD4 and CD8 (Fig. 1). PET scan showed irregular hypermetabolic soft tissue density shadows in nasal cavity and nasopharynx. She received radiotherapy from May 2020 to June 15th 2020 at local hospital. After radiotherapy, she complained of ocular symptoms. Then She took ophthalmic examination at local hospital. Her best-corrected visual acuity (BCVA) was 20/50 in the right eye and 20/20 in the left eye. There was anterior chamber inflammation in the right eye. Fundus examination of right eye was not clear, but normal at that time (Fig. 2). Ultrasonic biological microscope (UBM) showed scattered punctate echoes of anterior chamber in the right eye with superior and inferior iris roots apposed to the intracorneal surface, and rough and thickened ciliary body echoes throughout the circumference. She was treated with topical steroids (1% prednisolone) at local hospital but her eye symptoms got worse. She was then referred to our outpatient clinic at July 20th.

**Fig. 3:**
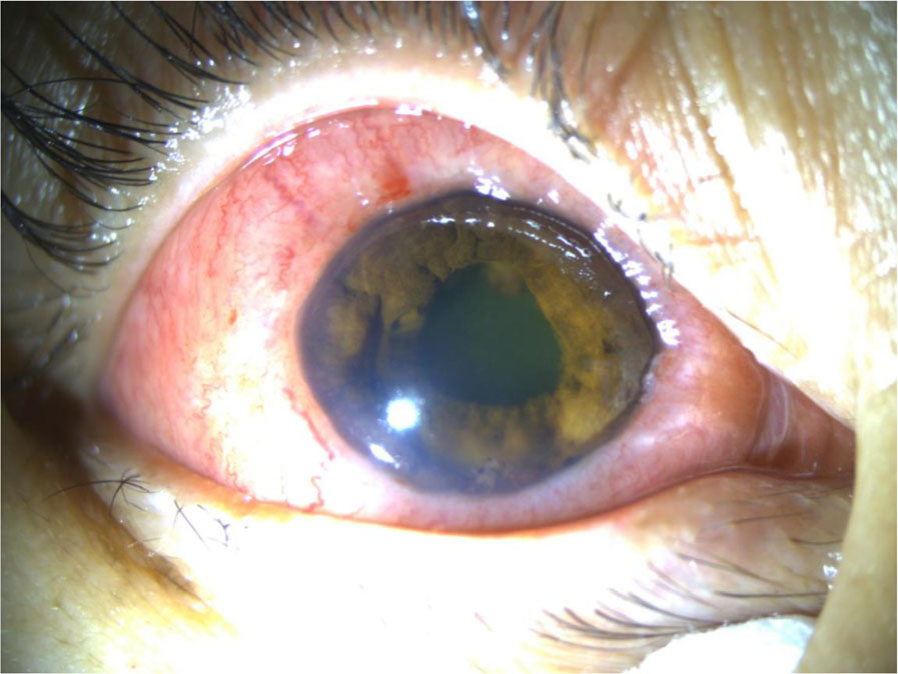
Anterior segment photography of right eye before diagnosis vitrectomy showed swelling conjunctiva, irregular ridgy iris and edge of mass in the vitreous cavity

**Fig. 4:**
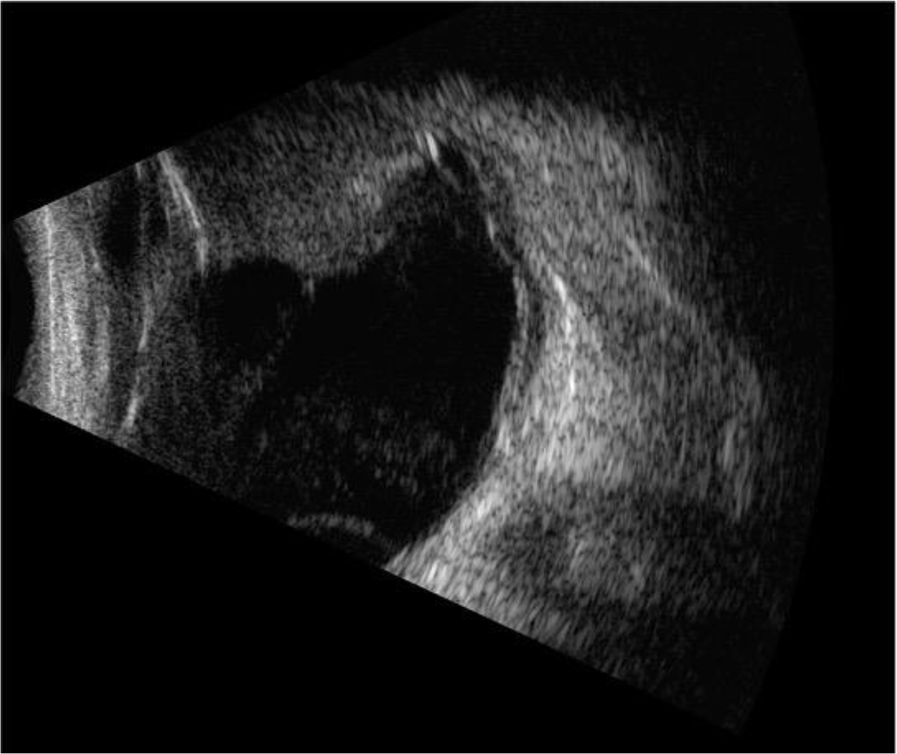
B-scan ultrasonograpy of right eye before diagnosis vitrectomy showed vitreous opacity and retinal detachment with ring occupying lesion of ciliary body

**Fig. 5:**
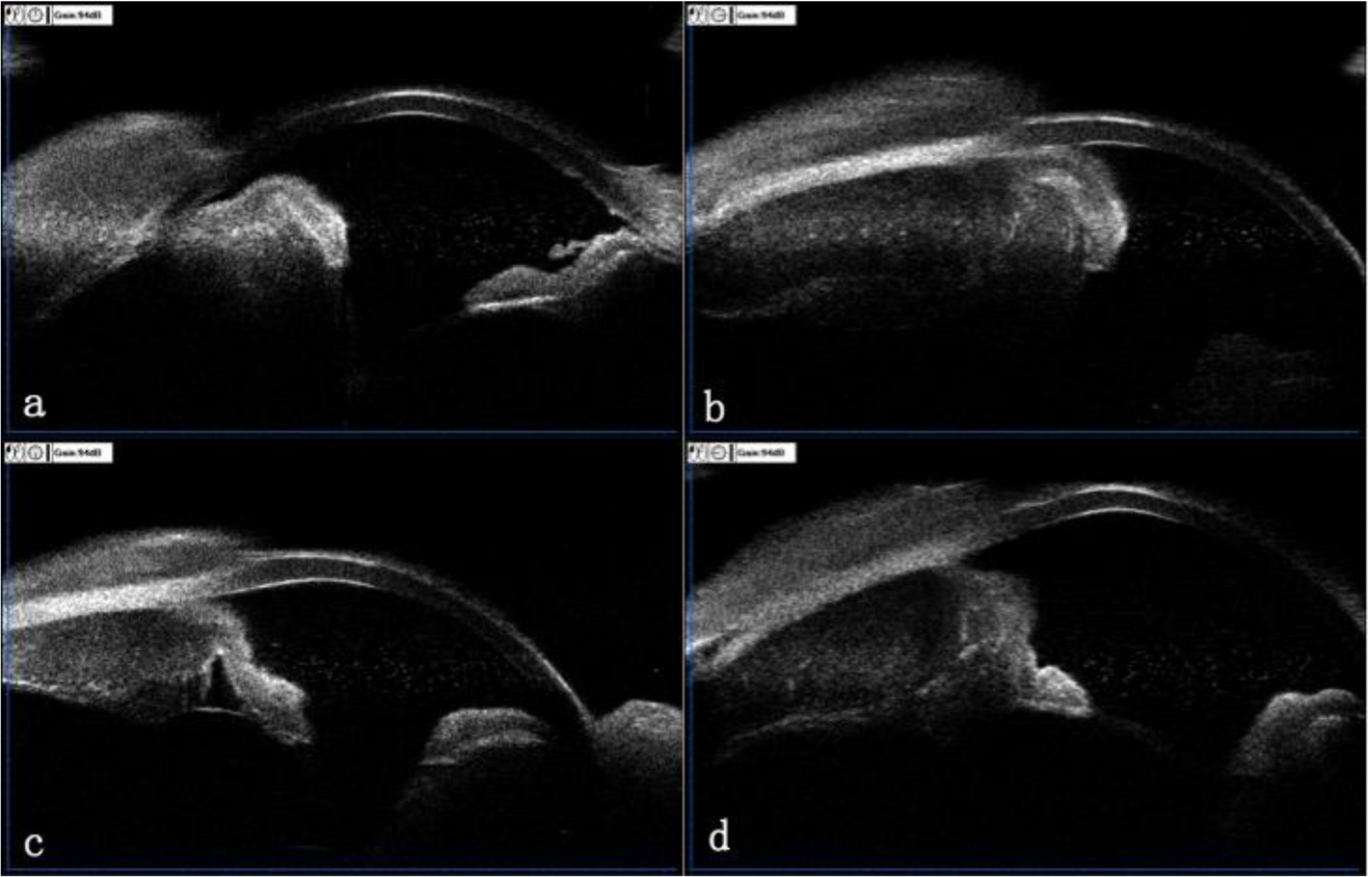
UBM of right eye before diagnosis vitrectomy of 12 o’clock(a), 3 o’clock(b), 6 o’clock(c) and 9 o’clock(d)

Ophthalmic examination revealed BCVA of finger count OD and 20/20 OS. Intraocular pressure was 29 mmHg in right eye and 18 mmHg in the left eye. The right conjunctiva showed injection and highly chemosis. There were grey-white keratic precipitations with anterior chamber cells and flares in the right eye. Her right iris was bulging in the nasal part with inferior local posterior synechia. Irregular pupil and opacity lens were shown in the right eye. The edge of a mass in the vitreous cavity was observed at the pupillary margin in the superior temporal region of right eye (Fig. 3). Dilated fundus examination of right eye revealed a serious yellowish-white vitreous opacity and the fundus situation was inaccessible. B-scan ultrasonography showed vitreous opacity and retinal detachment with ring occupying lesion of ciliary body in the right eye (Fig. 4). UBM showed the conjunctiva of the right eye was thickened, especially supranasal part, a 360 degree medium and low signal in the ciliary body, the thickest part was located at supranasal part. The iris was squeezed and deformed, suggesting a mass in the conjunctiva and ciliary body of the right eye (Fig. 5).

**Fig. 6:**
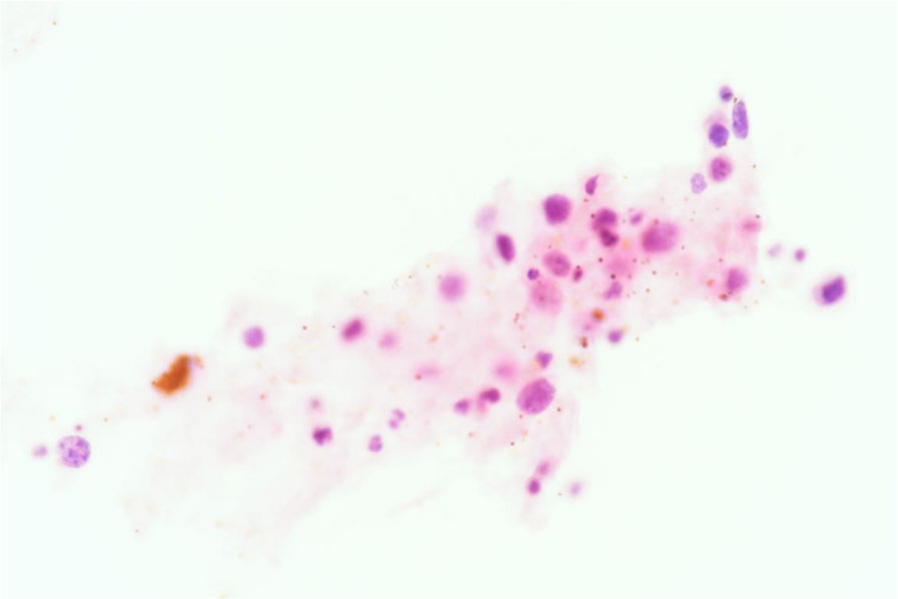
Photomicrograph of vitreous biopsy (hematoxylin-erosin; × 40) showed small malignant cells

**Fig. 7:**
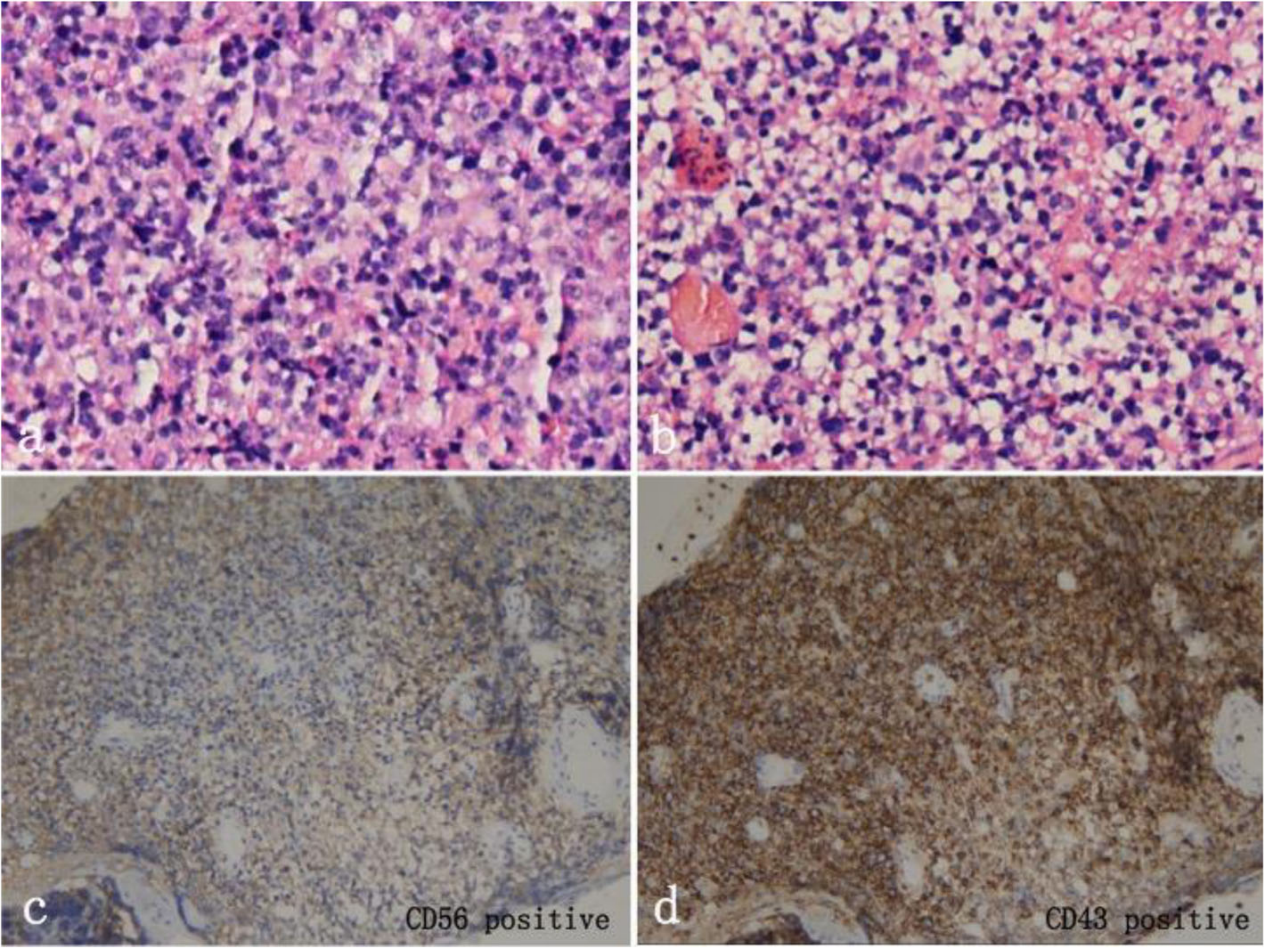
Histological and immunohistochemical images of conjunctiva biopsy. Immunohistology was positive for CD3, CD56, CD43, CD2, Ki67 85%, TIA1, CD4, and granzyme B, but negative for CD5, CD7, CD8, CD20 and CD5. Hematoxylin-erosin staining showed small-sized malignant cells (a, b). Immunostaining was positive for CD56 (c) and CD43 (d)

On July 27th, 2020, the patient underwent diagnostic vitrectomy with vitreous and conjunctiva biopsy of her right eye. Intraoperatively, the tissue under conjunctiva showed fish-like thickening. After removing the opacity vitreous, there was exudative retinal detachment without retinal tear. The cytological pathological diagnosis of vitreous specimen showed few dysplastic round cells with T-cell gene rearrangement (Fig. 6). The sample of vitreous contained high levels of IL-6(> 10,000 pg/ml), IL8(781.219 pg/ml), IL10(63.3 pg/ml) and VEGF (3264.677 pg/ml), but negative in EBV DNA. The in situ hybridisation staining of conjunctiva showed positive of EBV expressed RNA (EBER ISH) and in situ hybridisation (positive control). Immunohistology of conjunctiva was positive for CD3, CD56, CD43, CD2, Ki67 85%, TIA1, CD4, and granzyme B, but negative for CD5, CD7, CD8, CD20 and CD5 (Fig. 7). According to the clinical manifestations and biopsy, a diagnosis of vitreous, ciliary body and conjunctiva involvement of extra nodal malignant NK/T-cell lymphoma was made.

Systemic workups, including blood test, orbital and brain enhanced magnetic resonance imaging, PET-CT, chest computed tomography, cerebrospinal fluid analysis was made. Blood test for EBV-DNA was positive of 500copies/ml. Cerebrospinal fluid contained elevated leucocyte of 14 × 10^6^/L (0–8), elevated CSF-protein of 0.56 g/L (0.15–0.45) and high levels of IL-6 (8.9 pg/ml, < 5.9), IL8(63 pg/ml, < 62), IL10(18.6 pg/ml, < 9.1). Except for right ocular and nasal cavity involvement, no extra-central nervous system involvement was shown in the imaging examinations.

**Fig. 8:**
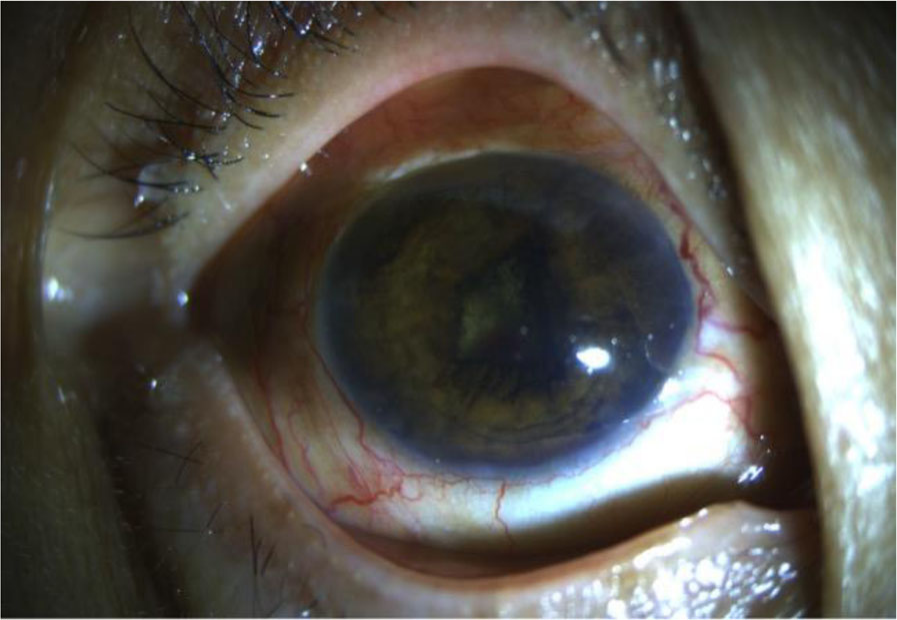
Anterior segment photography of right eye 3 month after diagnosis vitrectomy

The patient received chemotherapy of SMILE protocol (Methotrexate (MTX) 2.0 g/m^2^, 3.5 g, iv, 4 h, d1; Dexamethasone 40 mg, iv, d2–4; Ifosfamide (IFO) 1 g/m2, 1.7 g, iv, d2–5; Etoposide (VP-16) 100 mg, iv, d2–4; L-asparaginase 3750 IU, im, d5), accompanied with intravitreal injection of 400 μg/0.1 ml of methotrexate twice a week during the first month, followed by weekly injections for 2 months and monthly injection for 9 months. After 16 injections and 2 cycles of SMILE protocol, her visual acuity was stabled at hand moving, the conjunctival injection and chemosis relieved significantly and anterior segment inflammation disappeared (Fig. 8). But the retinal detachment still present.

## Discussion

ENKTL is a form of peripheral T cell lymphoma (PTCL), which is a group of generally aggressive neoplasms that constitute less than 15% of all non-Hodgkin lymphomas (NHL) [1, 2]. We experienced a rare case of nasal ENKTL related to ocular including conjunctiva, ciliary body, vitreous and retina. To the best of our knowledge, there have been several case reports and case series of NKTL with ocular involvement published [3–14], and our case was the first one which reported nasal NKTL with involvement of both intraocular with ciliary body related and orbit soft tissue.

In our case, the characteristics of the patient were dramatic deterioration of vision, highly injected and chemosis conjunctiva, ciliary body mass, uveitis and retinal detachment. Her ocular symptoms happened after the radiotherapy of nasal ENKTL and progress rapidly. The diagnosis was confirmed by biopsy of conjunctiva and vitreous specimen and systemic and local chemotherapy was initiated as soon as possible. Unfortunately, her visual acuity was not elevated after the therapy because of rapid progression of the disease, but her IOP decreased to normal, the chemosis conjunctiva and anterior chamber inflammation considerably reduced, the ciliary body mass shrank, no contralateral eye related or other organ involvement was identified and condition was stable 3 months after chemotherapy.

Since ENKTL has an aggressive clinical course and poor prognosis, its early diagnosis is of great significance for prolonging patents’ survival rate. Prognosis of ENKTL is mostly related to the stage and location of disease at diagnosis. Prognostic index of natural killer lymphoma (PINK-E) is a prognostic index for ENKTL treated with non-anthracycline-based chemotherapy [15]. In the retrospective analysis, the adverse prognostic factors were identified as following: age > 60 years, stage III or stage IV disease, distant lymph node involvement, non-nasal type disease and EBV-DNA positive. Each risk factor account for one point and patients of ENKTL were stratified by PINK-E into three categories: low risk (0 to 1 point), intermediate risk (2 points) and High risk (≥3 points). Three-year overall survivals were 25%, 62% and 81% of high, intermediate and low risk categories separately. Our patient’s PINK-E score was 1 point, which was low risk and had a relatively superior prognosis.

The atypical cells of ENKTL in immunophenotype express CD2, CD56, and cytoplasmic CD3, but do not express surface CD3. Most cases express cytotoxic granule proteins such as granzyme B, TIA-1, and perforin [16, 17]. Uncommon cases may express CD4, CD8, and/or CD7 [18–20]. In our case, the immunohistology of nasal biopsy was positive for CD3, CD56, Ki67 80%, TIA1, granzyme B and Epstein-Barr virus (EBV), but negative for CD20, PAX5, CD30, T-bet, CD4 and CD8. Immunohistology of conjunctiva was positive for CD3, CD56, CD2, Ki67 85%, TIA1, CD4, and granzyme B, but negative for CD5, CD7, CD8, CD20 and CD5. The immunophenotype of conjunctiva and previous nasal biopsy were both positive for CD3, CD 56, Ki 67, TIA1 and granzyme B, which were typical immunophenotype of ENKTL. The vitreous specimen showed atypical cells with T-cell gene rearrangement. These pathological findings confirmed the diagnosis of ENKTL related to ocular tissue.

Treatments of ENKTL include local irradiation and systemic chemotherapy. For intraocular ENKTL, ocular treatment of intravitreal MTX injection or globe radiotherapy was considered. Our patient received sinus radiation therapy at local hospital. After the ENKTL related to ocular tissue, we started systemic chemotherapy of SMILE procedure (methotrexate, dexamethasone, ifosfamide, etoposide and L-asparaginase) and local chemotherapy of intravitreal MTX injection (400 μg/0.1 ml). Numerous retrospective studies showed that outcomes after combined therapy (chemotherapy plus radiation therapy) are superior to radiation therapy alone or chemotherapy alone [21–23].

Our patient is undergoing systemic and local chemotherapy at present, and her ocular symptom is relieved significantly. But the major limitation of our case is the patient’s exudative retina detachment still present and her vision acuity impaired significantly. As ENKTL is an aggressive disease with a tendency to invade tissues and metastasize to close location, it is important to observe ocular related and begin aggressive combined therapy as early as possible.

## Data Availability

All data generated or analyzed during this study are included in the published article (and its supplementary information files).

## Abbreviations

ENKTL: Extranodal natural killer/T-cell lymphoma
NK: Natural killer
EBV: Epstein-Barr virus
BCVA: Best-corrected visual acuity
UBM: Ultrasonic biological microscope
MTX: Methotrexate
IFO: Ifosfamide
VP-16: Etoposide
PTCL: Peripheral T cell lymphoma
NHL: Non-Hodgkin lymphomas
PINK-E: Prognostic index of natural killer lymphoma
